# Study on the dynamic changes and formation pathways of metabolites during the fermentation of black waxy rice wine

**DOI:** 10.1002/fsn3.1507

**Published:** 2020-03-27

**Authors:** Li Jiang, Yingchun Mu, Su Wei, Yu Mu, Chi Zhao

**Affiliations:** ^1^ School of Liquor and Food Engineering Guizhou University Guizhou China

**Keywords:** black waxy rice wine, fermentation, metabolomics, multivariate statistical analysis

## Abstract

Black waxy rice wine fermentation metabolites are closely related to the product's final quality. However, little is known about dynamic metabolite changes during fermentation. Here, we used gas chromatography time‐of‐flight mass spectrometry (GC‐TOF‐MS) metabolomics and multivariate statistical analysis to explore the relationship between metabolites and fermentation time. A total of 159 metabolites were identified during the entire fermentation process. The PCA analysis revealed a clear separation between the samples after 4 days and 2 days, and the samples after 4–24 days clustered together. This indicated that BGRW fermentation progresses rapidly in the first 48 hr of fermentation. A total of 40 metabolites were identified as differential during fermentation (VIP > 1 and *p* < .05), including 12 organic acids, four amino acids, one fatty acid, 17 sugars and sugar alcohols, one alcohol, and five other metabolites. Pathway analysis showed that the differential metabolites were involved in 28 metabolic pathways, and the most commonly influenced pathways (impact value > 0.1 and *p* < .05) were galactose metabolism, pyruvate metabolism; starch and sucrose metabolism; alanine, aspartic acid, and glutamate metabolism; the tricarboxylic acid cycle, glyoxylic acid, and dicarboxylic acid metabolism; and amino sugar and nucleotide sugar metabolism. Moreover, the integrated metabolic pathway was generated to understand the transformation and accumulation of differential metabolites. Overall, these results provide a comprehensive overview of metabolite changes during black waxy rice wine fermentation.

## Graphical abstract

Based on metabonomic method, the changes and formation pathways of differential metabolites during the fermentation of black waxy rice wine were studied.

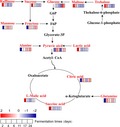

## 1 INTRODUCTION

Chinese rice wine is one of the oldest beverages in the world and has a history of more than 5,000 years (Yu, Ding, & Ye, 2012). Black waxy rice wine is a low‐alcohol wine made from black waxy rice, which is a local specialty of Guizhou, China. This wine has a unique flavor and high nutritional value, including an abundance of oligosaccharides, peptides, amino acids, vitamins, and minerals, and is widely consumed (Meng et al., 2016; Wei et al., 2016). As the local famous wine, people often offer it for hospitality. Prior research has shown that black waxy rice wine has the effect of tonifying the kidney (Qi, Li, Mu, Qiu, & Su, 2018). The brewing process of this wine is similar to Korean rice wine and mainly includes saccharification and alcoholic fermentation (Son, Lee, Kim, Seo, & Kim, 2018). Saccharification involves the breakdown of proteins in the raw materials by enzymes such as amylase and glucose amylase. During alcohol fermentation, yeast can produce ethanol, carbon dioxide, and so on (Hye‐Jung, Sang, Sang, & Young‐Suk, 2013; Kim, Kim, Bai, & Ahn, 2011). However, the metabolites produced in these two stages, such as free sugars (glucose and fructose), organic acids (lactic and acetic acids), and other flavoring compounds (mannitol and amino acids), are strongly related to the formation of flavor (Sang, Lee, Ji, Choi, & Che, 2013).

Metabolomics is a new science and technology that refers to a holistic analytical approach to all low‐molecular‐weight metabolites in an organism or a cell (Rochat & Bertrand, 2012). Conventional metabolomics techniques include nuclear magnetic resonance (NMR) spectroscopy, gas chromatography‐mass spectrometry (GC‐MS), and liquid chromatography‐mass spectrometry (LC‐MS) (Wishart, 2008). Compared with other analytical methods (e.g., LC‐MS and NMR spectroscopy), GC‐MS has the advantages of low cost, good reproducibility, high resolution, and small matrix effect (Kopka, 2006). In recent years, this method has been widely used in various food control, food genomics, and nutritional metabolomic applications (Christian et al., 2008; Khakimov, Bak, & Engelsen, 2014; Khakimov et al., 2016; Murgia, Scano, Cacciabue, Dessì, & Cabonia, 2019; Zeng et al., 2019). Furthermore, metabolomic studies based on gas chromatography time‐of‐flight mass spectrometry (GC‐TOF‐MS) have also been widely used to discover differences between samples (Hao et al., 2018; Yu et al., 2015). Therefore, the GC‐TOF‐MS method might potentially be the most suitable choice for the primary analysis of black waxy rice wine, considering the integrity and reliability of this approach.

At present, the dynamic changes and differences in metabolites during the brewing of this wine have not been reported. Therefore, the purpose of this paper is to comprehensively describe the changes in metabolites in the fermentation process of black waxy rice wine by GC‐TOF‐MS technology and to explore the differences in metabolites during brewing. The research results are helpful for understanding the flavor formation mechanism of black waxy rice wine and provide a scientific basis for guiding the production of the wine.

## 2 MATERIALS METHODS

### 2.1 Sample preparation

Fermentation starters (fermented by Z‐20 *Rhizopus*) used in this experiment were purchased from Huishui County Yonghong Winery, Guizhou Province, China. First, 20 kg of black waxy rice was washed and soaked in water overnight at room temperature and then steamed at 100°C for 1 hr. After the steamed rice was cooled to room temperature, it was mixed with fermentation starters (10 g) and transferred to wine jars for saccharification. The saccharification time is 2–3 days, and the temperature is required to be 25–28°C. In the end, sterile water was added to a 1:4 ratio of material and water, and the tank was sealed for fermentation. Triplicate independent brewing was conducted.

Fermented mash collected during the brewing time (2 days, 4 days, 6 days, 11 days, 17 days, and 24 days) was put into a sterile tube, sealed, and stored at −80℃ until further analysis.

### 2.2 Chemicals

HPLC‐grade methanol, chloroform, and pyridine were purchased from CNW Technologies and Adamas Reagent Co., Ltd., respectively. Methoxy amination hydrochloride was purchased from Tokyo Chemical Industry Co., Ltd. Ribitol was purchased from Sigma‐Aldrich trading Co., Ltd. BSTFA (with 1% TMCS, v/v) was purchased from Regis Technologies, Inc. Saturated fatty acid methyl esters (FAMEs) were purchased from Dr. Ehrenstorfer. All other chemicals and reagents used in this research were of analytical grade.

### 2.3 Metabolite extraction

Approximately 100 μl of each sample was transferred into 1.5‐ml Eppendorf (EP) tubes, followed by 300 μl of methanol extraction liquid and 5 μl of ribitol as an internal standard to the sample. The tubes were vortexed for 30 s and ultrasonicated for 10 min (in ice‐water baths). After centrifugation for 15 min at 14,825 *g* and 4°C, 30 μl of the supernatant was carefully pipetted into a 1.5‐ml EP tube, and 10 μl of each sample was mixed into a QC sample. Finally, the metabolite was dried in a vacuum concentrator and then extracted. Approximately 100 μl of Methoxyamination hydrochloride (20mg/mL in pyridine) methicinium reagent was added to the dried metabolite and incubated in an oven at 80°C for 30 min after mixing. Subsequently, 100 μl of BSTFA (containing 1% TMCS, v/v) was added to each sample, and the mixture was incubated at 70°C for 1.5 hr. When the sample was cooled to room temperature, 5 μl of FAMEs (dissolved in chloroform) was added.

### 2.4 GC‐TOF‐MS conditions

GC‐TOF‐MS analysis was performed using an Agilent 7,890 gas chromatograph system coupled with a Pegasus HT time‐of‐flight mass spectrometer. The system utilized a DB‐5MS capillary column coated with 5% diphenyl cross‐linked with 95% dimethylpolysiloxane (30 m × 250 μm inner diameter, 0.25 μm film thickness; J & W Scientific). A 1 μL aliquot of the analyte was injected in splitless mode. Helium was used as the carrier gas, the front inlet purge flow was 3 ml/min, and the gas flow rate through the column was 1 ml/min. The initial temperature was kept at 50°C for 1 min, increased to 310°C at a rate of 10°C/min, and then kept for 8 min at 310°C. The injection port, transfer line, and ion source temperatures were 280, 280, and 250°C, respectively. The energy was −70 eV in electron impact mode. The mass spectrometry data were acquired in full‐scan mode with an m/z range of 50–500 at a rate of 12.5 spectra per second after a solvent delay of 6.25 min.

### 2.5 Data processing and statistical analysis

The obtained metabolomic data were analyzed by the method of Kind et al. (2009). Chroma TOF 4.3 × software and LECO‐Fiehn Rt × 5 databases of LECO Corporation were used for raw peak extraction, data baseline filtration, baseline calibration, peak alignment, deconvolution analysis, peak identification, and integration of the peak area. Afterward, peaks detected in < 50% of QC samples or with RSD > 30% in QC samples were removed (Dunn et al., 2011). For relative quantification, all filtered data were normalized by the internal standard.

The data (sample name, standardized data of normalized peak area) were input into SIMCA 14.1 software (V14.1, MKS Data Analytics Solutions) for multivariate statistical analysis. PCA showed the distribution of raw data. OPLS‐DA can obtain sample separation. To estimate the stability and prediction ability of the OPLS‐DA model, the intercept values of *R* ^2^ and *Q* ^2^ are obtained through 200 permutations to verify whether the OPLS‐DA model is useful. The OPLS‐DA model was used to confirm the differential metabolites by combining the VIP projection value (VIP ≥ 1.0) and Student's *t*‐test (*p* < .05). Finally, MetaboAnalyst was used for pathway analysis (http://www.metaboanalyst.ca).

## 3 RESULTS AND DISCUSSION

### 3.1 Sample quality control and overall analysis of metabolites

In the total ion chromatogram (TIC), the ordinate indicated the peak intensity, and each peak represented one or more metabolites. The higher the peak intensity is, the higher the content of the substance is. The abscissa is the retention time (RT), which is the time it takes for the substance to reach its maximum concentration from the start of the injection to the passage through the column. The longer the time is, the longer it takes for the material to separate from the capillary column.

**Figure 1:**
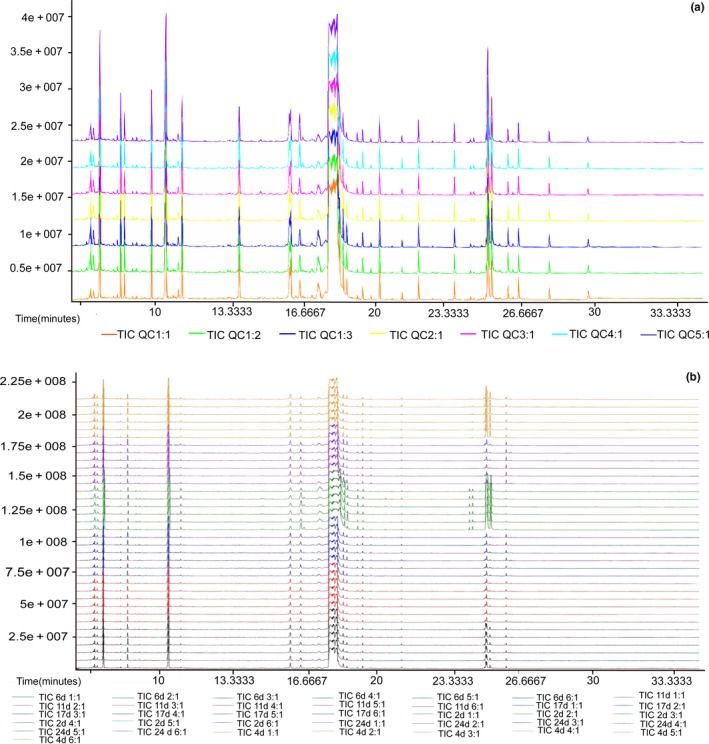
GC‐TOF‐MS and QC TIC of all samples. The ordinate shows the relative mass abundance, and the abscissa indicates the retention time

By observing the TIC of the QC sample (Figure 1a), it was found that the RTs and peak areas overlapped well, indicating that the instrument was stable and had a good stable signal when the same sample was detected at different times. In the TIC of all samples, no drift of the peaks was observed, showing steady RTs (Figure 1b). Therefore, the TIC can directly reflect the difference in metabolite profiles between samples.

In this experiment, a total of 468 peaks were monitored by GC‐TOF‐MS. The RT index of the peak detected by the instrument was compared with the value of the Fiehn database. If the difference between the RT value of the measured substance and the RT value of the database is within ± 5,000, the measured peak value is considered to be meaningful for the substance (Parker et al., 2015). A total of 159 metabolites were detected in these samples, and according to their chemical structure characteristics, they were divided into ten categories, which included 32 organic acids, 20 amino acids, 25 fatty acids, 27 sugars and sugar derivatives, six amines, five esters, eight alcohols, 10 ketones, three phenols, and 23 other metabolites (Table S1).

### 3.2 Multivariate statistical analysis

**Figure 2:**
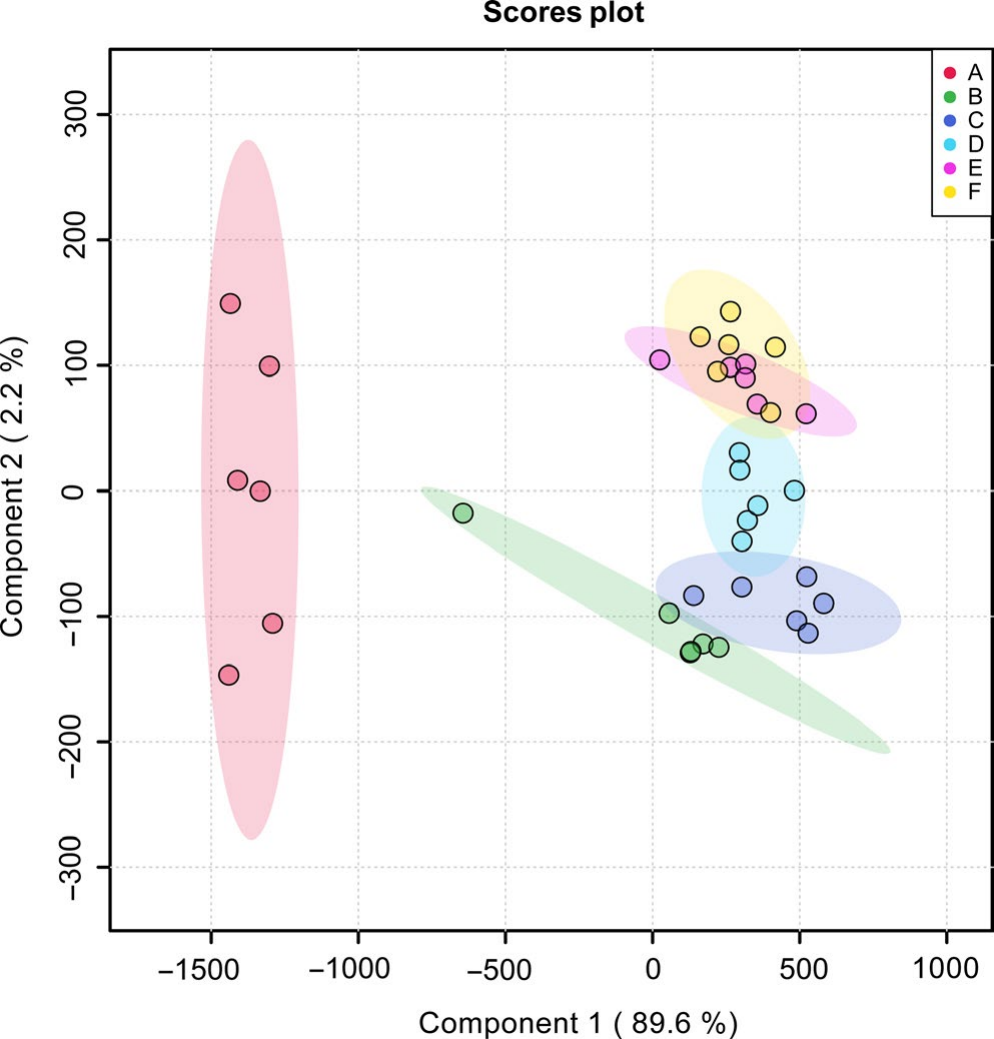
Metabolite PCA map of samples (colored ellipses indicate 95% confidence intervals for metabolites at each stage, a – 2 days; b – 4 days; c‐6 days; d – 11 days; e – 17 days; f – 24 days)

**Figure 3:**
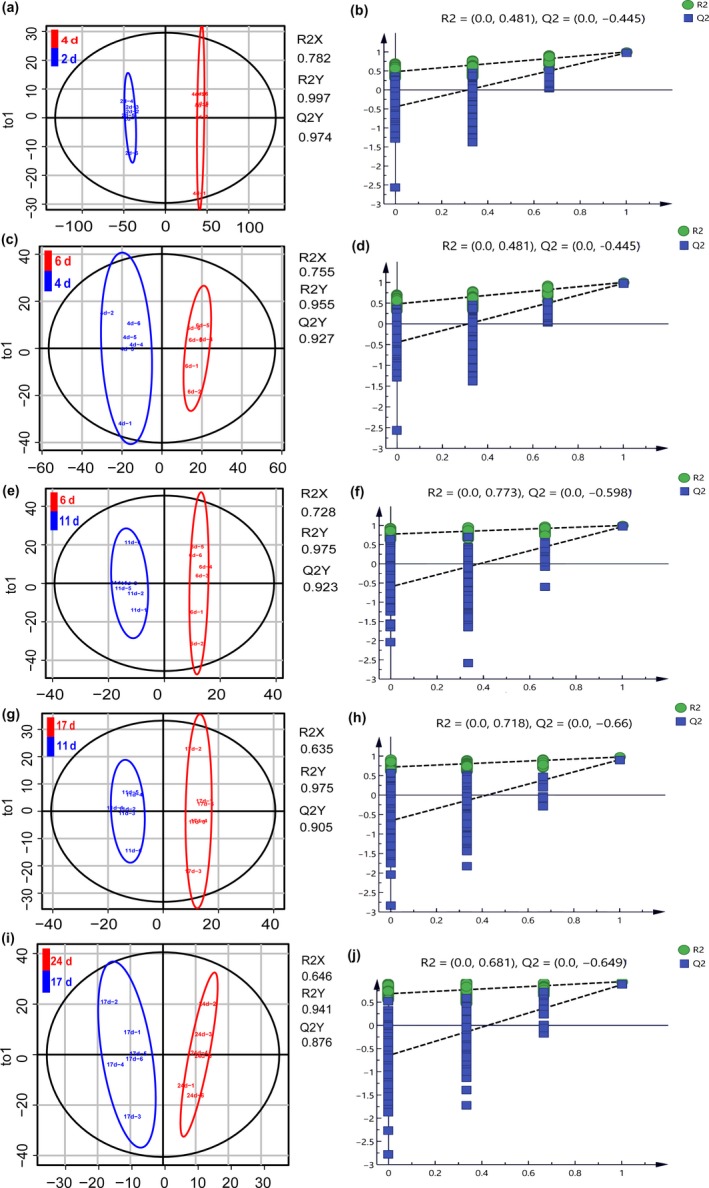
Orthogonal partial minimum discriminant analysis scores of the OPLS‐DA model (a, F‐2 days vs 4 days; b, G‐4 days vs 6 days; c, H‐6 days vs 11 days; d, I‐11 days vs 17 days; e, J‐17 days vs 24 days) and cross‐validation

Multivariate statistical method mainly through chromatography to distinguish and classify the measured variables to understand patterns and identify molecular markers (Johan, Elaine, & Torbj Rn, 2007; Kasote et al., 2014; Xia, Wu, & Yuan, 2007). Principal component analysis (PCA) and orthogonal partial least squares discriminant analysis (OPLS‐DA) are common metabolomic analysis tools. As shown in Figure 2, the unsupervised PCA model showed that the contribution rate of the two principal components was 91.8% (PC1, 89.6% and PC2, 2.2%). Moreover, all samples in the score plots were within the 95% Hotelling's T‐squared ellipse, which illustrated that no outlier existed among the analyzed samples. It is noteworthy that the metabolites after 4 days to 24 days of fermentation were clustered together, while the metabolites after 2 days were individually clustered into one class. Based on this result, we preliminarily considered that metabolites changed significantly after 2 days of fermentation, which is consistent with other reports (Suganuma, Fujita, & Kitahara, 2007). This significant metabolic change may be due to the favorable temperature and sufficient oxygen content of the fermentation samples during the saccharification period, which activates the microbial community in the fermented mash and results in rapid metabolite changes. To explore the major differential metabolites between adjacent phases, a supervised OPLS‐DA model was used to distinguish the samples. The score plots (Figure 3a,c,e,g and i) showed significant separation between the two stages. The R^2^Y and Q^2^ values were used to evaluate the stability of the model and explain the reliability of the original data. When the RY^2^ and *Q* ^2^ values are close to 1, it indicates that the model is stable (Lee, Jayaprakasha, Avila, Crosby, & Patil, 2019). Here, taking Figure 3a, for example, the RY^2^ and *Q* ^2^ values were 0.997 and 0.974, respectively, comparing 2 days to 4 days, suggesting that our model construction was successful. To avoid overfitting of the OPLS‐DA model, the permutation test was used for verification. The cross‐validation results are shown after 200 permutations (Figure 3b,d,f,h, and j). Once again taking Figure 3b as an example, the R^2^ and *Q* ^2^ values were 0.481 and −0.445, respectively; the low value of the *Q* ^2^ intercept indicated the stability of the model, revealing that the OPLS‐DA model has no overfitting. Consequently, this model was deemed suitable to explore the differences in the fermentation process in this study.

### 3.3 Differential metabolite analysis

**Figure 4:**
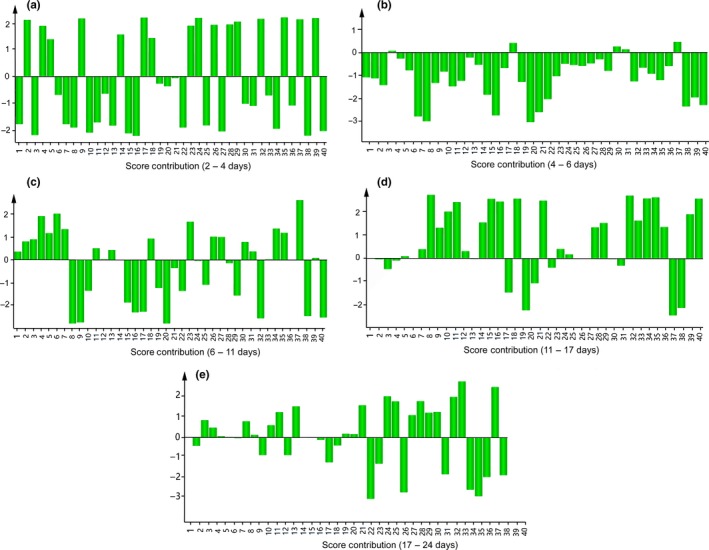
Adjacent phase metabolite contribution map (a, 2 days ‐4 days; b, 4 days ‐6 days; c, 6 days – 11 days; d, 11 days ‐17 days; e,17 days ‐24 days)

Based on the results of OPLS‐DA, a total of 40 different metabolites (VIP > 1 and *p* < .05) were identified during the fermentation process (Table S2). Among them, 12 organic acids, four amino acids, 1 fatty acid, 17 sugars and sugar alcohols, one alcohol, and five other metabolites were included. Figure 4 shows the contribution of differential metabolites at each fermentation stage. From 2 days to 4 days of fermentation, myo‐inositol (no. 34), fructose (no. 16), uracil (no. 23), isomaltose (no. 38), pyruvic acid (no. 8), citric acid (no. 31), glutathione (no. 36), N‐cyclohexylformamide (no. 2), 3,6‐anhydro‐D‐galactose (no. 28), etc., were found to be the metabolites contributing to changes (Figure 4a). However, we observed few changes from 4 days to 6 days of fermentation (Figure 4b). Glutathione (no. 36), 2,4‐diaminobutyric acid (no. 6), alanine (no. 4), glycine (no. 22), sorbitol (no. 33), conduritol b epoxide (no. 7), etc., were significantly responsible for changes from 6 days to 11 days (Figure 4c). Furthermore, pyruvic acid (no. 8), citric acid (no. 31), myo‐inositol (no. 3), sorbitol (no. 33), glucose (no. 18), raffinose (no. 39), sophorose (no. 15), gentiobiose (no. 21), fructose (no. 16), α‐D‐glucosamine 1‐phosphate (no. 11), etc., were found to be the metabolites that mainly contributed to differentiation from 11 days to 17 days (Figure 4d). Finally, myo‐inositol (no. 3), isomaltose (no. 38), oxoproline (no. 25), sorbitol (no. 25), 2‐deoxy‐D‐galactose (no. 29), maleamate (no. 26), glycine (no. 22), etc., were considered to be the metabolites contributing to changes after fermentation from 17 days to 24 days (Figure 4e).

**Figure 5:**
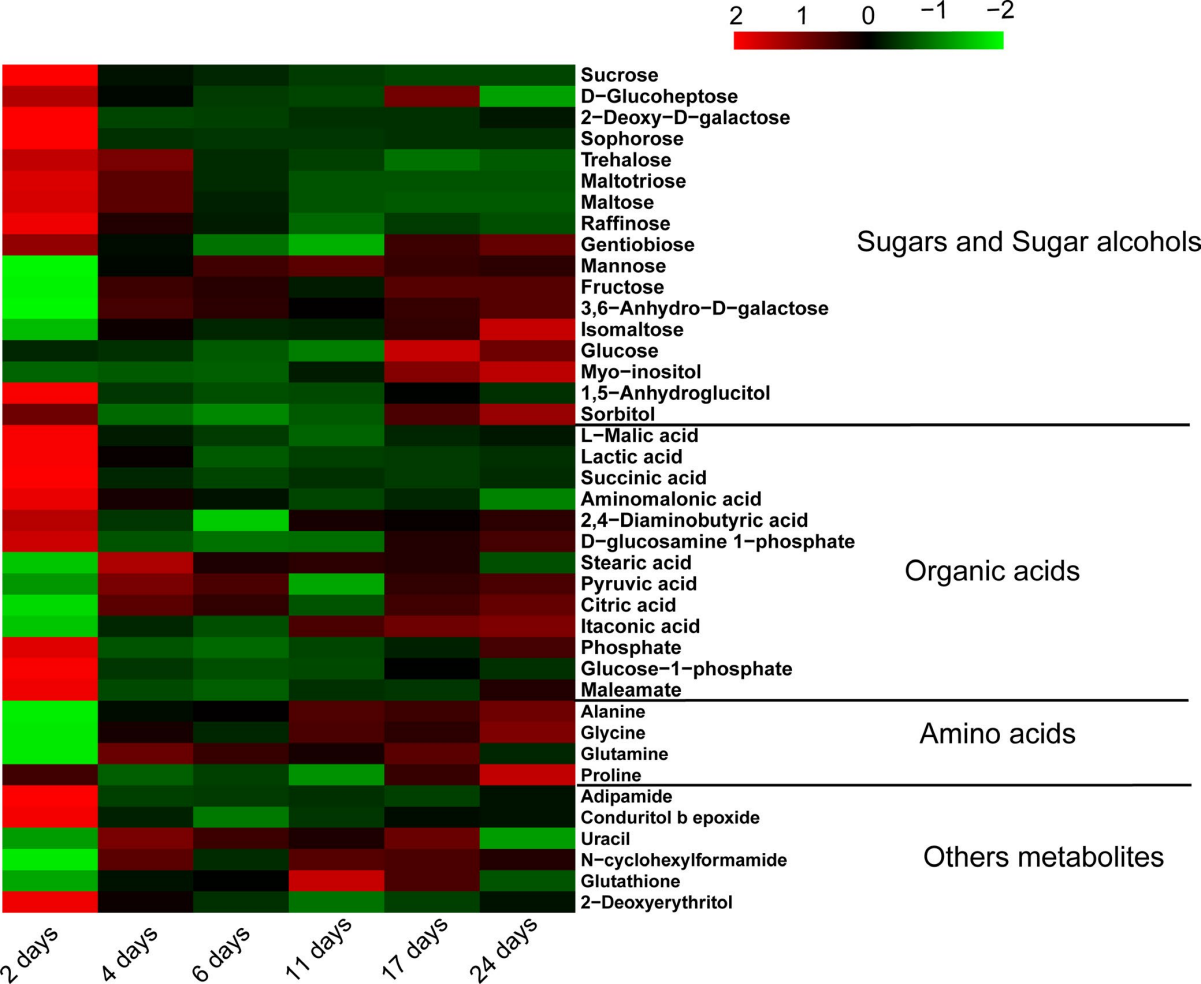
The relative content of differential metabolites in black waxy rice wine at different fermentation times

The relative content of 40 different metabolites was used to plot the heat map for intuitively discovering the changes in differential metabolites during fermentation (Figure 5). The red and green represent the above‐ and below‐average metabolites, respectively.

Fluctuating changes in sugars were observed throughout fermentation. This is because the starch in the raw material is hydrolyzed to form sugar, which enters the tricarboxylic acid cycle through the glycolysis pathway during the fermentation to produce a compound such as an organic acid (Klosowski et al., 2015). At the same time, sugar can also provide energy for the growth of microorganisms through carbohydrate metabolism pathways (Da et al., 2016). In the early stage of fermentation, the content of maltotriose, sucrose, maltose, trehalose, gentiobiose, 2‐deoxy‐D‐galactose, D‐glucopyranose, sucrose, and raffinose increased. However, the levels of these sugars showed a decreasing trend after 4 days of fermentation, especially sophorose, 2‐deoxy‐d‐galactose, and sucrose, which were significantly decreased (*p* < .05). The reason for the declining sucrose content may be that sucrose is directly metabolized by microorganisms or hydrolyzed by hydrolytic enzymes to produce fructose and glucose during fermentation (Liu, Hsu, Lee, & Liao, 2009). Meanwhile, the content of fructose and 3,6‐anhydro‐d‐galactose increased after 4 days of fermentation, and glucose levels increased after 17 days. Trehalose can be used not only as an important storage carbon source, but also as a protective agent to enhance cellular components against adverse conditions (Kim et al., 2011). Maltose is a disaccharide, and the main reason for its lower content is that amylases and glucose amylases constantly degrade maltose into glucose as fermentation progresses. In addition, isomaltose, a disaccharide with two glucose molecules linked by an α‐1,6 glycosidic bond, was detected in this study. Isomaltose is not used by yeast in fermentation, so the content gradually increases in the fermentation process and reaches the highest level after 24 days. The content of mannose increased significantly after 2 days of fermentation (*p* < .05) and reached its highest level after 11 days but began to decrease after 17 days. Based on this result, we suspect that in the late stage of fermentation, mannose will be phosphorylated to form mannose‐6‐phosphate due to the action of hexokinase, resulting in a decrease in the content of this sugar.

Interestingly, sugar alcohols such as 1,5‐anhydroglucitol, D‐sorbitol, and inositol were also detected in this work. However, a relatively large change in content was observed for inositol, increasing in the later stage of fermentation. Inositol is an essential component of cell membranes and plays an important role in cell growth and brain function. As a basis for several signaling molecules and secondary messenger molecules, inositol is involved in many biological processes (Hye‐Jung et al., 2010).

According to reports, organic acids are the main components that affect the taste of fermented alcoholic beverages, such as sake (Ding, Suzuki, & Koizumi, 1995; Kodama et al., 2002), and can also inhibit bacteria during the brewing process. We found that 13 organic acids were differential metabolites. The content of L‐malic acid, lactic acid, succinic acid, aminomalonic acid, 2,4‐diaminobutyric acid, α‐d‐glucosamine 1‐phosphate, phosphate, glucose‐1‐phosphoric acid, and maleamate was highest at 2 days of fermentation. In contrast, as the fermentation proceeded, the contents of L‐malic acid, succinic acid, α‐d‐glucosamine 1‐phosphate, phosphate, glucose‐1‐phosphoric acid, and maleamate were decreased significantly (*p* < .05). Lactic acid is produced by the action of pyruvate through lactate dehydrogenase, and the content decreased after 4 days of fermentation. We suspected that this may be the reaction of lactic acid with alcohol produced by yeast fermentation to form ethyl lactate. It is known that phosphate is a principal intermediate metabolite involved in signal transduction pathway regulation. The level of phosphate decreases after 2 days of fermentation for two reasons. First, large amounts of ADP phosphorylation occur in the glycolysis pathway and TCA cycle (Son et al. 2018). Second, some phosphoric acid was used to activate proteins that regulate signal transduction pathways to adapt to the environment. Citric acid, L‐malic acid, and succinic acid are intermediates of glycolysis and the TCA cycle (Gibbs & Bowser, 2009). The content of citric acid was the highest after 24 days of fermentation. Previous studies have shown that the formation of citric acid can limit the TCA cycle. The content of malic acid and succinic acid changed similarly and remained relatively stable after 4 days of fermentation. Stearic acid, as a saturated fatty acid, has a higher content after 4 days; it is mainly involved in fatty acid metabolism and has a significant impact on the flavor of rice wine (Xiao, Dai, Zhu, & Yu, 2015). However, pyruvate levels increased after 4 days of fermentation and then began to decline after 6 days. Pyruvate is a key intermediate product of several metabolic pathways and can be converted to carbohydrates via gluconeogenesis, to fatty acids or energy through acetyl CoA, and to amino acids and ethanol.

Amino acids are the precursors for the generation of higher alcohols, aldehydes, esters, and ketoacids (Hye et al. 2010), contributing to the sensory properties of wine. In this study, alanine, glycine, proline, and glutamine were identified as differential metabolites. It has been reported that the detection of amino acid prefermentation may be mainly caused by the introduction of fermentation starters and the production of proteases and peptidases produced by microorganisms in the fermented mash, which hydrolyze the proteins in the raw materials (Kim et al., 2011). However, the increase in amino acid content during postfermentation may be mainly caused by the dissolution of amino acids in microbial cells. Alanine and glycine are sweet amino acids (Su et al., 2014), and their levels began to increase after 2 days of fermentation. After 24 days of fermentation, the glycine content was approximately two times higher than at the initial stage of fermentation, and the alanine content was approximately five times higher. Alanine has important physiological functions as a neurotransmitter or hormone regulator to control metabolism (Wu, Gibbs, & Farb, 1993) and as an intermediate metabolite of various active substances (coenzyme A, pantothenic acid, etc.) in the body (White, Gunyuzlu, & Toyn, 2001). Alanine also helps resist fatigue and senility and improves exercise ability and memory (Hobson, Saunders, Ball, Harris, & Sale, 2012). The proline content showed a downward trend after 4 days of fermentation and then increased to the end of fermentation. As the most important amino acid in mammalian protein synthesis, proline has special effects on protein structure and thermal stability. It accounts for 1/3 of amino acids in collagen, which accounts for 30% of the protein in the body (Liu et al., 2018). Additionally, glutamine contributes to the catabolism and anabolism of nitrogen and is one of the main sources of intracellular nitrogen (Mora, 1990). The content of glutamine increased rapidly after 4 days of fermentation but decreased after 6 days, which might be particularly related to the consumption of nitrogen at the end of the fermentation. According to the reported, the main reason for the decrease in amino acid content is the Strecker reaction (Zhang et al., 2017).

In addition, 2‐deoxyerythritol, n‐cyclohexylformamide, conduritol b epoxide, adipamide, and glutathione were observed. 2‐Deoxyerythritol is produced by DL‐malic acid through the Ehrlich pathway, and the content was highest after 2 days of fermentation and lowest after 17 days. Glutathione is an active tripeptide composed of glutamate, cysteine, and glycine with essential physiological functions (Kritzinger, Bauer, & du Toit, 2013). It is involved in the tricarboxylic acid cycle and glucose metabolism. In the winemaking process, glutathione was produced in large quantities after 11 days of fermentation and subsequently converted to other substances, leading to a decrease in content.

### 3.4 Metabolic pathway analysis

**Figure 6:**
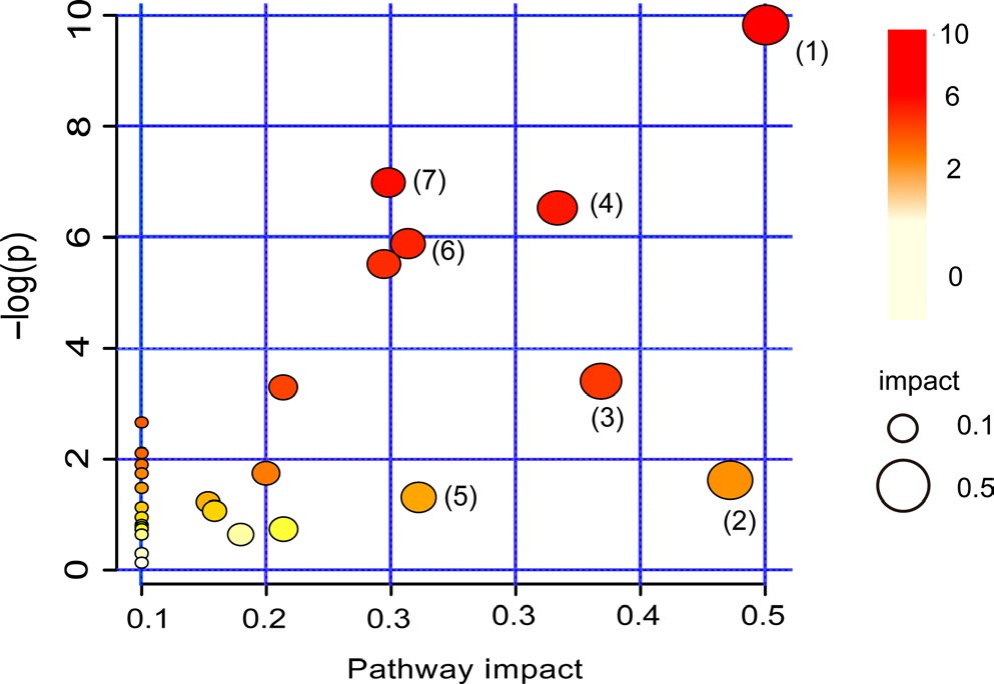
Enriched bubble diagram of differential metabolite pathways (bubble size is proportional to the degree of influence of each pathway; bubble color indicates the significant degree of influence, from the highest (red) to the lowest (white))

To explore the potential metabolic pathway of black waxy rice wine at the fermentation stage, MetaboAnalyst 4.0 was used to analyze the enrichment of differential metabolites. Although the *p*‐value calculated by the enrichment analysis indicates the enrichment degree of the metabolic pathway, there is no standard threshold for it (Wang et al., 2018). As shown in Figure 6, the differential metabolites are involved in 28 metabolic pathways (Table S3). The impact value and the *p*‐value (impact value > 0.1 and *p* < .05) were applied as the criteria to explore the key metabolic pathways (Chen et al., 2018), and 7 key metabolic pathways were found. These pathways were galactose metabolism (1); pyruvate metabolism (2); starch and sucrose metabolism (3); alanine, aspartic, and glutamate metabolism (4); the tricarboxylic acid cycle (5), glycolic acid, and dicarboxylic acid metabolism (6); and amino sugar and nucleotide sugar metabolism (7). However, the surprise is that this result was similar to that of Ferdouse et al. (2018).

**Figure 7:**
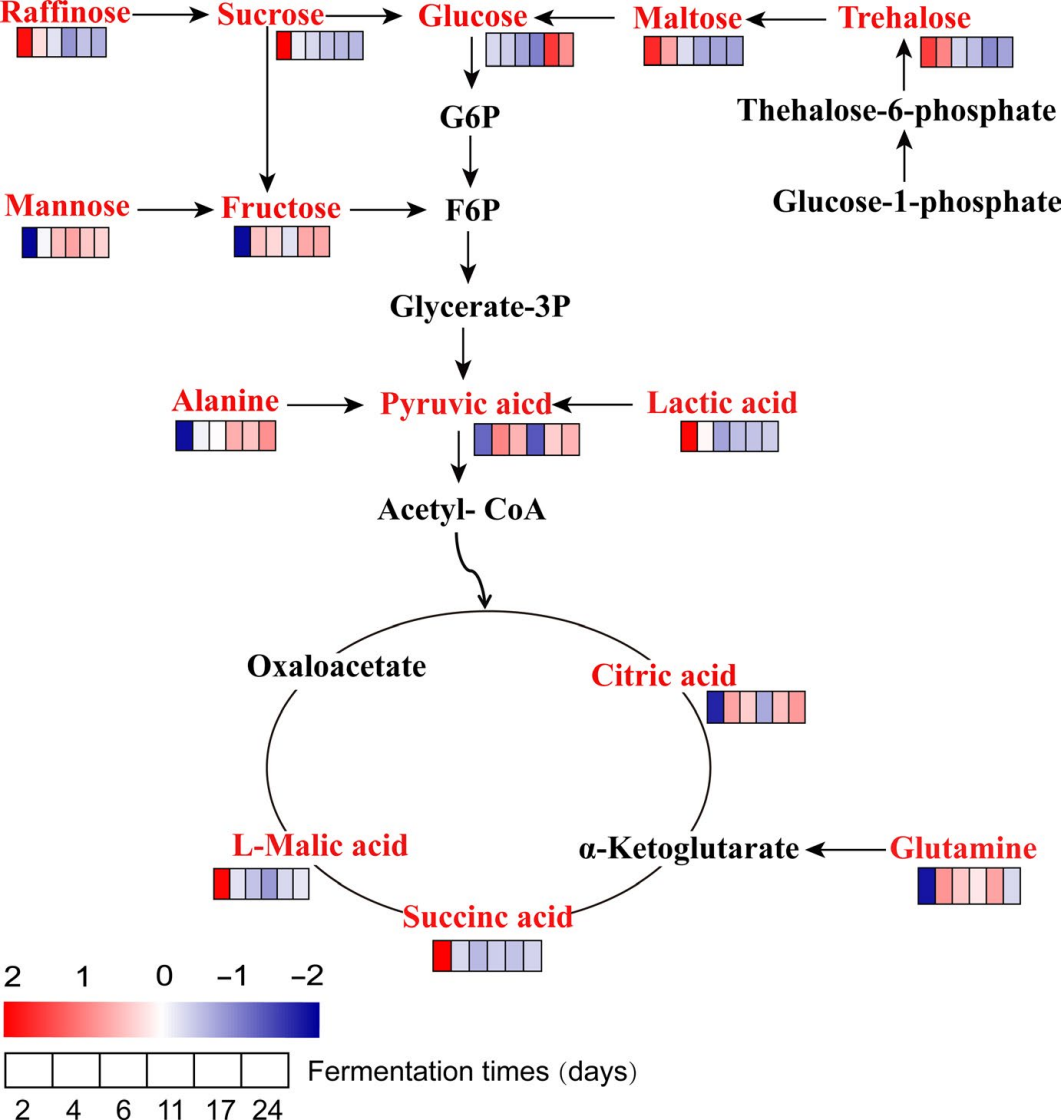
Metabolic pathway analysis of the black waxy rice wine fermentation process (F6p, fructose 6‐phosphate; G6p, glucose 6‐phosphate; glycerate‐3P, glycerate‐3 phosphate)

For the relationship between the metabolic pathways and differential metabolites (Figure 7), red and black represent differential metabolites and intermediates, respectively. A comprehensive analysis of the metabolites and metabolic pathways at different fermentation times revealed that 7 key metabolic pathways were related to glucose‐1‐phosphate, pyruvic acid, L‐malic acid, lactic acid, succinic acid, alanine, sucrose, glucose, raffinose, fructose, maltose, mannose, trehalose, and glutamine.

On the one hand, the content of raffinose, maltose, trehalose, and sucrose decreased during fermentation. On the other hand, the glucose and fructose levels increased. This observation may be because in the galactose metabolic pathway, raffinose produces sucrose under the action of galactosidase, and then, sucrose is converted to glucose and fructose by the action of invertase. In addition to starch sucrose metabolism, glucose‐1‐phosphate (G1P) under the activity of G1P transferase and trehalose 6‐phosphate (T6P) synthase creates T6P. Then, T6P is activated by trehalose‐6‐phosphate phosphatase (TPP) to produce trehalose. Trehalose reacts with maltose α‐d‐glucan transferase to form maltose. Finally, glucose is produced under the action of maltose phosphorylase.

Organic acids such as citric acid, L‐malic acid, and succinic acid are products of the TCA cycle, playing an essential role in the flavor formation of wine, and are called "wine skeletons" (Zhang et al., 2017). During fermentation, glucose is subjected to hexokinase in the glycolytic pathway to produce G6P, subsequently producing F6P under the action of isomerase. F6P reacts with a series of enzymes to form pyruvate and is then oxidized and decarboxylated to form acetyl CoA, thus entering the TCA cycle. The same lactic acid can also form oxaloacetate chloride in the TCA cycle under the action of lactate dehydrogenase (Figure 7). In addition, pyruvate can enter the amino acid metabolic pathway through the amino acid transamination reaction, providing a carbon skeleton for amino acid synthesis, and can be oxidized and decarboxylated to form acetyl CoA.

## 4 CONCLUSIONS

In summary, the GC‐TOF‐MS‐based metabolomic approach was applied to assess metabolite changes during traditional black waxy rice wine fermentation to investigate the relationship between metabolites and fermentation time. The metabolites changed significantly in the first 2 days of the entire fermentation process. Analysis of further integrated key metabolic pathways showed that galactose; pyruvate; starch and sucrose; alanine, aspartic acid, and glutamate; the tricarboxylic acid cycle, glyoxylic acid, and dicarboxylic acid; and amino sugar and nucleotide sugar metabolisms were the most important metabolic pathways during fermentation. This present study is the first to identify differential metabolites and their formation pathways in the traditional black waxy rice wine fermentation process.

## CONFLICT OF INTEREST

The authors declare that they have no conflict of interest.

## AUTHOR CONTRIBUTIONS

Li Jiang was responsible for designing the experiments and writing the manuscript. Yingchun Mu (corresponding author) conducted the experimental design and revised the manuscript. Wei Su helped collating experimental data. Yu Mu and Chi Zhao helped to collect samples, as well as post‐processing.

## ETHICAL APPROVAL

This study does not involve any human or animal testing.

## Supporting information

Table S1‐S3
