# Supplementary material for: Study on the dynamic changes and formation pathways of metabolites during the fermentation of black waxy rice wine
Source: Food Sci Nutr. 2020 Mar 27;8(5):2288–98. doi: 10.1002/fsn3.1507 (PMC7215209; doi:10.1002/fsn3.1507)
Supplement: Supplementary file 1 — Table S1‐S3 [file FSN3-8-2288-s001.docx]

**Supplemental Table 1** **The relative concentrations of metabolites during fermentation of black waxy rice wine**

| Compound | Similarity | R.T. (minutes) | Mass | 2d | 4d | 6d | 11d | 17d | 24d |
| --- | --- | --- | --- | --- | --- | --- | --- | --- | --- |
| 2-hydroxypyridine | 883.05 | 7.212 | 152 | 358.87±17.59 | 336.82±12.86 | 322.29±15.78 | 347.87±27.93 | 352.73±27.19 | 348.66±19.23 |
| Pyruvic acid | 913.59 | 7.347 | 174 | 2.69±0.93 | 16.37±0.9 | 14.08±1.56 | 1.85±0.17 | 12.92±1.02 | 14.07±0.93 |
| lactic acid | 807.90 | 7.521 | 117 | 3776.64±129.57 | 2703.81±336.71 | 2273.52±190.14 | 2389.72±87.18 | 2412.16±178.16 | 2439.96±118.62 |
| 2-ketobutyric acid | 376.96 | 7.598 | 221 | 5.17±0.22 | 3.24±1.36 | 4.04±0.36 | 2.72±1.99 | 3.53±1.62 | 3.36±1.51 |
| glycolic acid | 914.97 | 7.719 | 66 | 18.11±1.04 | 8.78±0.52 | 8.21±0.34 | 8.27±0.63 | 8.61±0.32 | 8.56±0.65 |
| Maleimide | 830.14 | 7.995 | 154 | 3.23±0.36 | 4.57±0.23 | 4.56±0.31 | 5.61±0.3 | 4.95±0.44 | 5.45±0.33 |
| alanine | 847.76 | 8.094 | 116 | 3.86±0.4 | 13.46±2.64 | 14.14±0.79 | 17.42±1.47 | 16.64±3.15 | 18.87±1.82 |
| hydroxylamine | 457.87 | 8.300 | 207 | 0.35±0.03 | 0.2±0.04 | 0.14±0.1 | 0.17±0.08 | 0.19±0.09 | 0.16±0.07 |
| oxalic acid | 331.16667 | 8.393 | 245 | 0.44±0.03 | 0.42±0.02 | 0.47±0.03 | 0.49±0.04 | 0.44±0.22 | 0.43±0.2 |
| 2-hydroxybutanoic acid | 482.55 | 8.402 | 73 | 7.45±0.96 | 7.58±1.05 | 9.52±0.41 | 9.85±1.57 | 8.31±1.49 | 9.58±0.85 |
| 2-Furoic Acid | 557.53 | 8.567 | 125 | 0.49±0.1 | 0.49±0.13 | 0.56±0.18 | 0.53±0.16 | 0.54±0.23 | 0.53±0.13 |
| 3-Hydroxypropionic acid 1 | 886.14 | 8.681 | 177 | 1.95±0.08 | 0.8±0.04 | 0.78±0.04 | 0.73±0.05 | 0.76±0.07 | 0.83±0.07 |
| 4-Hydroxypyridine | 847.59 | 8.830 | 152 | 8.98±0.98 | 9.21±0.78 | 9.02±0.55 | 9.39±0.89 | 9.35±0.58 | 9.24±0.8 |
| glutaraldehyde | 468.53 | 8.867 | 86 | 0±0 | 7.03±1.31 | 6.8±1.46 | 8.67±0.76 | 9.54±1.27 | 11.57±1.15 |
| 3-hydroxybutyric acid | 682.50 | 8.903 | 147 | 5.31±0.22 | 3.52±0.39 | 3.27±0.42 | 2.52±0.09 | 2.89±0.31 | 3.27±0.19 |
| sulfuric acid | 456.88 | 9.001 | 281 | 55.55±1.95 | 32.66±1.73 | 32.48±2.22 | 34.13±1.36 | 33.79±2.27 | 35.13±2.42 |
| Lactamide | 280.87 | 9.045 | 75 | 1.45±0.71 | 3.66±0.37 | 2.65±0.28 | 3.6±0.4 | 2.71±0.25 | 3.11±0.31 |
| cycloleucine | 304.00 | 9.505 | 57 | 2.13±2.15 | 4.5±0.4 | 4.21±0.62 | 4.72±0.55 | 4.57±0.24 | 4.29±0.4 |
| malonic acid | 670.86 | 9.539 | 103 | 2.22±0.33 | 1.29±0.09 | 1.41±0.25 | 1.31±0.08 | 1.19±0.56 | 1.08±0.5 |
| valine | 830.38 | 9.685 | 144 | 0.00 | 3.75±0.42 | 3.36±0.58 | 3.58±0.43 | 4.35±1.18 | 4.73±0.66 |
| canavanine degr prod | 356.13 | 9.721 | 156 | 0.00 | 1.14±0.11 | 1.19±0.16 | 1.39±0.13 | 1.49±0.29 | 1.36±0.11 |
| 2-Butyne-1,4-diol | 637.13 | 9.875 | 147 | 4.71±0.25 | 1.91±0.13 | 1.79±0.06 | 1.7±0.15 | 1.72±0.26 | 1.77±0.14 |
| hydroxyurea | 452.72 | 9.979 | 147 | 4.11±1.85 | 1.49±1.16 | 2.41±0.35 | 2.03±0.91 | 0.00 | 0.00 |
| 4-hydroxybutyrate | 553.29 | 10.04 | 117 | 9.25±0.66 | 3.31±0.21 | 2.81±0.26 | 2.12±0.15 | 2.66±0.2 | 2.95±0.15 |
| benzoic acid | 800.17 | 10.18 | 105 | 2.38±0.19 | 2.63±0.25 | 2.43±0.17 | 2.68±0.36 | 2.42±0.29 | 2.35±0.2 |
| Dihydroxyacetone | 640.55 | 10.19 | 103 | 3.32±0.28 | 1.81±0.14 | 1.61±0.21 | 1.81±0.14 | 1.64±0.11 | 1.83±0.09 |
| Carbobenzyloxy-L-leucine degr1 | 229.05 | 10.202 | 57 | 23.6±1.56 | 27.97±1.01 | 25.3±3.56 | 28.28±3.06 | 26.64±1.73 | 25.78±2.44 |
| phosphate | 839.60 | 10.463 | 299 | 481.37±41.84 | 354.69±8.36 | 345.7±21.23 | 362.25±17.16 | 376.36±21.81 | 419.22±29.74 |
| thymidine | 201.10 | 10.540 | 341 | 0.00 | 0.14±0.13 | 0.16±0.16 | 0.08±0.18 | 0.13±0.19 | 0.17±0.17 |
| 3-hydroxypyruvate | 375.45 | 10.585 | 226 | 0.00 | 0.13±0.17 | 0.24±0.18 | 0.24±0.24 | 0.00 | 0.44±0.03 |
| N-cyclohexylformamide | 279.90 | 10.612 | 57 | 105.3±4.98 | 156.61±6.41 | 135.23±18.41 | 155.62±12.09 | 153.61±7.51 | 147.44±8.5 |
| 2-Deoxyerythritol | 920.60 | 10.722 | 117 | 50.17±2.62 | 32.21±1.16 | 27.45±2.42 | 22.17±1.2 | 26.12±2.29 | 29.81±1.25 |
| Isoleucine | 595.16 | 10.783 | 158 | 0.00 | 0.79±0.69 | 0.68±0.59 | 1.2±0.3 | 1.23±0.72 | 2.29±0.36 |
| maleic acid | 297.46 | 10.861 | 341 | 2.89±0.28 | 2.16±0.08 | 2.17±0.25 | 2.35±0.22 | 2.28±0.26 | 2.28±0.14 |
| glycine | 874.24 | 10.968 | 174 | 16.3±2.18 | 24.71±0.73 | 22.8±1.7 | 26.52±1.3 | 25.52±1.22 | 28.11±1.33 |
| succinic acid | 872.79 | 11.073 | 147 | 567.72±24.84 | 254.4±11.07 | 225.68±18.15 | 248.11±13.74 | 235.98±14.08 | 252.1±11.69 |
| 1,3-Cyclohexanedione | 203.25 | 11.198 | 341 | 1.12±0.1 | 0.00 | 0.00 | 0.44±0.44 | 0.00 | 0.00 |
| D-Glyceric acid | 814.56 | 11.269 | 189 | 5.86±0.29 | 2.47±0.13 | 2.37±0.07 | 2.64±0.11 | 2.61±0.22 | 2.92±0.19 |
| uracil | 715.19 | 11.387 | 99 | 0.00 | 4.84±0.38 | 3.76±1.71 | 3.29±2.33 | 4.63±0.61 | 0.00 |
| Itaconic acid | 287.00 | 11.443 | 207 | 7.84±0.73 | 13.83±2.96 | 12.3±1.5 | 17.94±4.69 | 19.34±2.19 | 19.92±0.73 |
| Pyrrole-2-Carboxylic Acid | 223.61 | 11.487 | 152 | 18.01±1.22 | 12.26±0.96 | 11.7±0.61 | 12.29±0.6 | 12.54±0.94 | 12.46±1.06 |
| fumaric acid | 857.71 | 11.570 | 245 | 9.08±0.29 | 3.81±0.21 | 3.61±0.33 | 2.23±0.1 | 3.73±0.26 | 3.92±0.17 |
| Citraconic acid | 453.58 | 11.621 | 221 | 1.81±0.21 | 0.91±0.41 | 0.95±0.08 | 1.03±0.09 | 0.83±0.27 | 0.95±0.15 |
| serine | 635.85 | 11.671 | 147 | 12.81±2.39 | 7.26±2.05 | 9.12±1.73 | 8.75±1.02 | 8.44±0.31 | 9.5±0.87 |
| 3-Hydroxynorvaline | 288.19 | 11.699 | 228 | 1.59±0.17 | 1.07±0.12 | 1.09±0.2 | 1.22±0.12 | 1.42±0.36 | 1.21±0.09 |
| Pelargonic acid | 753.12 | 11.728 | 117 | 2.01±0.53 | 1.91±0.85 | 1.96±0.53 | 2.52±0.77 | 2.17±0.39 | 1.94±0.8 |
| Pipecolinic acid | 276.31 | 11.825 | 57 | 1.81±0.2 | 2.34±0.17 | 1.95±0.33 | 2.15±0.19 | 2.23±0.31 | 2.03±0.21 |
| 3-Methylamino-1,2-propanediol | 427.44 | 11.911 | 117 | 13.06±1.45 | 3.54±0.19 | 3.12±0.34 | 2.72±0.15 | 2.96±0.2 | 3.17±0.12 |
| resorcinol | 297.03 | 12.012 | 240 | 4.13±1.87 | 3.6±1.54 | 4.43±0.31 | 4.76±0.57 | 4.22±0.54 | 4.3±0.62 |
| L-Allothreonine | 565.38 | 12.028 | 69 | 8.39±1.45 | 5.9±0.64 | 4.81±0.38 | 5.56±0.7 | 5.29±0.19 | 5.4±0.76 |
| 1-Indanone | 237.79 | 12.075 | 143 | 2.63±0.21 | 1.05±0.47 | 1.62±0.42 | 1.41±0.24 | 1.31±0.6 | 1.21±0.21 |
| Glutaric Acid | 359.53 | 12.203 | 305 | 0.53±0.12 | 0.23±0.03 | 0.25±0.05 | 0.24±0.04 | 0.2±0.1 | 0.16±0.12 |
| 1,2-Cyclohexanedione | 414.06 | 12.263 | 138 | 0.00 | 6.34±0.34 | 5.75±0.53 | 4.95±0.3 | 5.25±0.41 | 5.67±0.25 |
| Biuret | 265.86 | 12.439 | 99 | 0.00 | 0.58±0.38 | 0.00 | 0.31±0.44 | 0.28±0.39 | 0.25±0.35 |
| beta-Alanine | 518.33 | 12.636 | 174 | 5±0.22 | 5.19±0.29 | 5.26±0.38 | 5.67±0.34 | 5.56±0.25 | 5.28±0.25 |
| 5-Methylresorcinol | 308.00 | 12.822 | 69 | 1.65±1.21 | 0.77±0.73 | 0.00 | 0.00 | 0.14±0.31 | 0.00 |
| L-Threose 1 | 480.08 | 12.828 | 147 | 6.06±2.76 | 4.51±1.98 | 5.68±0.25 | 5.46±0.45 | 5.6±0.55 | 5.55±0.28 |
| 2,4-diaminobutyric acid | 316.16 | 12.935 | 156 | 39.81±4.18 | 34.06±4.14 | 30.38±0.33 | 35.94±2.4 | 35.6±3.87 | 36.44±3.31 |
| 3-Aminoisobutyric acid | 543.31 | 12.996 | 86 | 8.71±0.79 | 9.44±0.78 | 9.28±1.15 | 10.25±0.84 | 9.71±0.66 | 9.75±0.62 |
| Aminomalonic acid | 354.29 | 13.132 | 69 | 14.88±1.1 | 10.03±1.16 | 9.07±0.76 | 7.79±0.82 | 8.52±1.67 | 6.37±0.66 |
| 3-hydroxy-L-proline | 338.00 | 13.1410 | 129 | 0.74±0.54 | 0.74±0.1 | 0.44±0.32 | 0.43±0.2 | 0.07±0.16 | 0.04±0.1 |
| L-Malic acid | 826.00 | 13.346 | 147 | 51.99±4.12 | 26.7±1.7 | 24.09±2.4 | 20.05±1.86 | 25.56±3.7 | 27.06±2.97 |
| Threitol | 413.94 | 13.447 | 305 | 12.47±5.63 | 3.15±1.32 | 2.55±1.81 | 2.06±2.08 | 4.73±0.4 | 5.02±0.42 |
| 4-Hydroxy-6-methyl-2-pyrone | 219.07 | 13.536 | 152 | 0.83±0.38 | 0.81±0.05 | 0.65±0.29 | 0.59±0.33 | 0.72±0.13 | 0.82±0.07 |
| aspartic acid | 728.94 | 13.754 | 232 | 0.00 | 1.42±0.86 | 2.28±1.06 | 2.61±0.44 | 2.69±0.77 | 4.03±0.27 |
| proline | 786.48 | 13.772 | 156 | 28.75±4.49 | 51.64±3.71 | 47.55±4.46 | 53.92±3.83 | 53.86±3.82 | 62.02±1.67 |
| Maleamate | 559.00 | 13.880 | 147 | 43.68±3.7 | 24.86±1.35 | 23.4±1.94 | 26.17±1.72 | 25.94±2.88 | 31.08±1.11 |
| 4-aminobutyric acid | 715.44 | 13.893 | 174 | 3.1±1.5 | 14.94±0.62 | 13.51±1.19 | 14.92±0.85 | 13.77±0.67 | 15.39±0.75 |
| Menthone | 282.59 | 14.126 | 69 | 2.17±0.98 | 3.24±0.3 | 2.74±0.47 | 2.41±1.17 | 2.69±1.28 | 2.82±0.43 |
| 2-aminophenol | 264.54 | 14.191 | 220 | 0.72±0.61 | 0.00 | 0.18±0.15 | 0.35±0.16 | 0.24±0.2 | 0.22±0.16 |
| Dodecanol | 213.83 | 14.248 | 71 | 4.43±0.46 | 5.39±0.83 | 4.5±0.52 | 4.91±0.6 | 5.16±0.64 | 4.62±0.6 |
| (2R,3S)-2-hydroxy-3-isopropylbutanedioic acid | 397.77 | 14.389 | 71 | 4.45±0.31 | 3.44±1.01 | 1.31±1.67 | 0.86±1.39 | 0.00 | 0.00 |
| 4-Hydroxyphenylethanol | 746.64 | 14.412 | 179 | 35.38±1.51 | 14.23±0.55 | 12.41±0.81 | 11.58±0.57 | 12.31±0.63 | 13.53±0.57 |
| 3-Phenyllactic acid | 741.37 | 14.539 | 193 | 2.46±1.11 | 2.34±0.08 | 1.9±0.12 | 1.9±0.12 | 1.86±0.16 | 1.99±0.08 |
| D-erythronolactone | 542.05 | 14.721 | 145 | 1.73±0.22 | 1.38±0.12 | 1.16±0.48 | 1.51±0.13 | 1.42±0.27 | 1.21±0.3 |
| Digitoxose | 521.46 | 14.812 | 204 | 12.31±5.9 | 9.83±0.83 | 7.3±1.52 | 9.01±2.14 | 7.22±0.51 | 9.06±1.74 |
| 4-Hydroxybenzoic acid | 242.60 | 14.978 | 207 | 3.66±0.35 | 3.48±0.36 | 3.2±0.49 | 3.43±0.48 | 3.82±0.58 | 3.09±0.19 |
| phenylalanine | 670.08 | 15.051 | 218 | 0.00 | 1.11±0.42 | 1.32±0.57 | 2.25±0.34 | 2.05±0.68 | 3.37±0.17 |
| trans,trans-Muconic acid | 325.74 | 15.075 | 229 | 0.07±0.06 | 0.65±0.25 | 0.51±0.24 | 0.65±0.33 | 1±0.18 | 0.66±0.3 |
| 3-ureidopropionate | 215.40 | 15.115 | 57 | 5.04±2.31 | 7.85±1.28 | 6.61±1.02 | 6.97±0.67 | 7.53±0.92 | 6.88±0.98 |
| Lyxose | 604.56 | 15.278 | 117 | 4.29±2.97 | 7.72±0.47 | 6.89±0.61 | 6.49±0.56 | 8.73±0.63 | 8.11±0.91 |
| ribose | 754.10 | 15.420 | 103 | 7.84±5.59 | 21.25±0.94 | 19.05±1.7 | 19.66±1.21 | 19.97±1.31 | 21.64±1.33 |
| Ribonic acid, gamma-lactone | 345.00 | 15.541 | 204 | 5.88±0.68 | 2±0.21 | 2.14±0.16 | 2.28±0.28 | 2.8±0.35 | 3.03±0.35 |
| Levoglucosan | 591.86 | 15.848 | 191 | 9.97±1.54 | 3.25±0.32 | 3.31±0.21 | 3.86±0.37 | 3.85±0.47 | 4.35±0.44 |
| xylitol | 605.56 | 15.912 | 103 | 0.00 | 1.82±0.17 | 1.42±0.65 | 1.77±0.12 | 2.18±0.76 | 1.9±0.23 |
| Acetol | 215.21 | 15.953 | 71 | 27.43±1.38 | 34.71±6.14 | 27.82±3.42 | 30.24±3.62 | 31.69±3.42 | 28.86±3.51 |
| glutamine | 303.90 | 15.997 | 154 | 15.5±2.15 | 62.65±11.02 | 55.05±11.34 | 50.77±6.5 | 60.23±2.76 | 42.69±4.04 |
| glycocyamine | 244.92 | 16.287 | 112 | 0.00 | 0.60±0.40 | 0.50±0.52 | 0.32±0.45 | 0.00 | 0.00 |
| putrescine | 788.27 | 16.318 | 174 | 0.00 | 3.01±0.09 | 3.58±0.28 | 3.85±0.19 | 4.66±0.37 | 5.66±0.31 |
| Diglycerol | 556.38 | 16.406 | 191 | 14.19±1.19 | 6.11±0.41 | 6.26±0.25 | 6.79±0.72 | 7.56±0.45 | 7.93±0.7 |
| citrulline | 383.69 | 16.437 | 85 | 18.27±1.03 | 15.38±10.79 | 18.81±2.22 | 16.87±7.67 | 21.56±0.79 | 19.43±2.02 |
| 3,6-Anhydro-D-galactose | 835.50 | 16.444 | 231 | 7.57±5.84 | 35.43±1.44 | 33.49±1.68 | 29.58±1.9 | 34.07±2.18 | 37.15±1.92 |
| D-(glycerol 1-phosphate) | 675.75 | 16.534 | 299 | 1.77±1.26 | 2.35±0.11 | 2.38±0.23 | 2.42±0.17 | 3.11±0.63 | 3.01±0.18 |
| Glucose-1-phosphate | 765.28 | 16.609 | 217 | 988.06±237.32 | 351.16±22.99 | 288.58±38.83 | 334.32±56.22 | 463.65±10.17 | 431.51±35.38 |
| 2-Deoxy-D-galactose | 554.81 | 16.758 | 218 | 96.45±67.34 | 7.95±0.76 | 9.44±3.4 | 14.22±4.27 | 13.63±1.79 | 21.37±4.35 |
| Adipamide | 320.09 | 17.081 | 231 | 31.95±20.72 | 3.52±1.17 | 4.04±1.17 | 5.23±1.88 | 3.96±1.62 | 7.83±2.96 |
| citric acid | 826.45 | 17.164 | 273 | 2.85±4.05 | 42.39±1.85 | 37.22±3.89 | 19.58±1.21 | 39.28±2.18 | 43.36±2.68 |
| alpha-D-glucosamine 1-phosphate | 409.25 | 17.192 | 193 | 29.52±13.38 | 17.95±0.45 | 16.68±0.98 | 16.87±0.63 | 22.76±1.49 | 24.16±1.33 |
| isocitric acid | 448.18 | 17.267 | 191 | 28.26±4.03 | 10.59±1.15 | 10.7±0.83 | 12.11±1.2 | 13.36±1.35 | 15.11±1.75 |
| 1,5-Anhydroglucitol | 630.00 | 17.459 | 244 | 43.69±30.13 | 15.8±2.78 | 13.37±1.4 | 13.85±3.11 | 20.58±2.42 | 16.21±1.13 |
| 3-Hydroxyanthranilic acid | 234.31 | 17.692 | 264 | 0.00 | 0.43±0.4 | 0.86±0.13 | 1.23±0.56 | 1.09±1.09 | 3.22±0.2 |
| fructose | 863.66 | 17.774 | 307 | 0.00 | 24.87±1.09 | 23.21±1.85 | 17.32±0.99 | 26.93±2.46 | 26.73±12.11 |
| mannose | 838.37 | 17.937 | 83 | 16.24±6.99 | 136.58±58.58 | 172.86±11.54 | 184.91±4.16 | 166.05±12.35 | 161.48±13.5 |
| glucose | 733.46 | 18.267 | 377 | 2.63±0.44 | 2.36±0.24 | 1.47±0.69 | 0.57±0.25 | 8.22±1.49 | 6.11±1.11 |
| allose | 256.79 | 18.211 | 343 | 0.76±0.36 | 5.01±1.44 | 5.55±2.57 | 2.69±2.88 | 4.77±2.35 | 4.16±1.54 |
| lysine | 426.31 | 18.332 | 156 | 0.00 | 1.18±0.16 | 1.42±0.73 | 1.64±0.33 | 2.09±0.47 | 2.95±0.11 |
| mannitol | 900.69 | 18.365 | 345 | 2.82±1.99 | 2.57±0.15 | 2.19±0.14 | 1.97±0.11 | 2.39±0.19 | 2.59±0.14 |
| sorbitol | 537.69 | 18.368 | 204 | 56.09±4.5 | 38.45±0.95 | 35.96±2.46 | 39.89±1.36 | 53.04±2.22 | 59.57±2.59 |
| D-galacturonic acid | 468.02 | 18.456 | 333 | 9.26±0.56 | 2.58±0.12 | 2.84±0.36 | 2.43±0.26 | 3.39±0.35 | 4.05±0.49 |
| tyrosine | 744.13 | 18.454 | 179 | 0.00 | 0.00 | 0.25±0.13 | 0.38±0.04 | 0.45±0.2 | 0.56±0.03 |
| conduritol b epoxide | 649.21 | 18.587 | 361 | 61.75±10.58 | 32.11±1.18 | 23.16±1.47 | 30.01±4.84 | 34.4±5.47 | 33.77±2.28 |
| 9-Fluorenone | 258.31 | 18.665 | 85 | 6.81±0.39 | 9.04±0.55 | 8.38±0.78 | 8.92±1.04 | 8.56±0.58 | 8.14±0.73 |
| lipoic acid | 210.05 | 18.821 | 71 | 1.44±0.1 | 2.08±0.25 | 1.76±0.37 | 1.9±0.28 | 1.64±0.39 | 1.82±0.43 |
| Galactonic acid | 868.05 | 18.984 | 333 | 1.98±0.43 | 1.11±0.26 | 1.21±0.37 | 1.74±0.45 | 0.98±0.22 | 1.3±0.3 |
| Saccharic acid | 244.09 | 19.045 | 293 | 0.21±0.1 | 0.26±0.12 | 0.25±0.12 | 0.79±0.36 | 0.14±0.13 | 0.23±0.03 |
| mucic acid | 352.82 | 19.447 | 219 | 0.44±0.06 | 0.43±0.12 | 0.3±0.05 | 0.39±0.13 | 0.34±0.13 | 0.33±0.11 |
| palmitic acid | 956.31 | 19.474 | 117 | 186.88±11.5 | 197.09±12.39 | 196.18±8.98 | 191.19±18.75 | 203.9±21.34 | 178.35±22.2 |
| N-Acetyl-D-galactosamine | 468.67 | 19.798 | 204 | 12.73±2.29 | 1.87±0.87 | 1.94±0.63 | 2.40±0.74 | 2.60±1.36 | 2.44±1.32 |
| myo-inositol | 916.14 | 19.851 | 217 | 10.58±0.3 | 31.37±0.57 | 28.79±2.18 | 31.98±1.41 | 45.17±1.87 | 57.84±2.5 |
| trans-3,5-Dimethoxy-4-hydroxycinnamaldehyde | 422.93 | 20.167 | 174 | 15.47±2.21 | 2.26±0.46 | 1.74±0.48 | 2.38±0.43 | 2.26±0.77 | 2.38±0.66 |
| caffeic acid | 279.56 | 20.292 | 204 | 0.00 | 0.64±0.03 | 0.38±0.28 | 0.54±0.05 | 0.84±0.2 | 1.02±0.09 |
| d-Glucoheptose | 569.74 | 20.5189 | 174 | 27.62±7.43 | 16.97±1.45 | 14.01±4.04 | 13.35±6.08 | 24.22±5.18 | 7.88±1.34 |
| glutathione | 349.38 | 20.551 | 67 | 0.25±0.19 | 2.06±0.13 | 2.28±0.33 | 4.79±0.36 | 3.22±0.27 | 1.28±0.25 |
| Phytol | 329.82 | 20.607 | 88 | 0.00 | 0.40±0.04 | 0.47±0.07 | 1.04±0.11 | 0.73±0.18 | 0.25±0.02 |
| Fructose 2,6-biphosphate degr prod | 335.07 | 20.761 | 101 | 0.08±0.08 | 0.21±0.09 | 0.20±0.10 | 0.19±0.1 | 0.33±0.08 | 0.29±0.06 |
| beta-Mannosylglycerate | 571.22 | 20.810 | 243 | 11.62±1.88 | 5.74±1.06 | 3.98±0.48 | 3.18±0.4 | 2.32±0.22 | 1.53±0.19 |
| linoleic acid | 399.14 | 20.978 | 67 | 1.01±0.04 | 0.23±0.16 | 0.26±0.12 | 0.54±0.26 | 0.46±0.08 | 0.00 |
| oleic acid | 422.823 | 21.031 | 117 | 0.85±0.04 | 0.38±0.17 | 0.30±0.18 | 0.46±0.1 | 0.29±0.14 | 0.00 |
| Elaidic acid | 351.11 | 21.200 | 131 | 2.23±0.16 | 3.24±0.89 | 2.66±0.3 | 2.71±0.27 | 2.91±0.43 | 2.28±0.25 |
| stearic acid | 932.72 | 21.2650 | 117 | 85.19±5.85 | 119.86±13.59 | 106.59±14.05 | 108.17±11.41 | 107.07±7.99 | 96.37±9.67 |
| Glucoheptonic acid | 471.29 | 21.493 | 73 | 7.68±0.68 | 3.18±1.36 | 3.32±1.57 | 5.34±0.31 | 3.53±1.60 | 5.61±2.65 |
| glucose-6-phosphate | 761.14 | 21.844 | 73 | 19.13±2.67 | 9.92±0.92 | 8.43±0.97 | 7.6±0.66 | 7.89±0.72 | 7.12±0.79 |
| Purine riboside | 717.71 | 22.267 | 217 | 9.72±0.44 | 2.85±0.09 | 2.55±0.16 | 2.71±0.21 | 2.74±0.28 | 2.74±0.25 |
| 1-Methyladenosine | 374.11 | 22.404 | 71 | 3.34±1.17 | 5.40±1.30 | 4.44±0.6 | 4.38±0.67 | 5.23±0.52 | 4.32±0.42 |
| 6-phosphogluconic acid | 635.79 | 22.657 | 318 | 8.17±0.35 | 5.32±0.36 | 5.75±0.52 | 6.44±0.37 | 6.30±0.25 | 6.10±0.47 |
| Arachidic acid | 548.05 | 22.919 | 117 | 0.57±0.09 | 0.94±0.28 | 0.63±0.3 | 0.75±0.08 | 0.76±0.23 | 0.72±0.07 |
| cytidine-monophosphate degr prod | 496.80 | 23.026 | 387 | 1.18±0.12 | 0.79±0.15 | 0.85±0.19 | 0.87±0.15 | 0.78±0.10 | 0.76±0.06 |
| DL-dihydrosphingosine | 673.14 | 23.176 | 204 | 0.15±0.09 | 1.69±0.24 | 1.48±0.19 | 1.31±0.15 | 1.43±0.13 | 1.54±0.16 |
| Neohesperidin | 369.52 | 23.215 | 69 | 1.44±0.22 | 0.88±0.27 | 0.74±0.19 | 0.56±0.06 | 0.50±0.28 | 0.30±0.14 |
| salicin | 324.71 | 23.476 | 57 | 1.04±0.17 | 1.18±0.27 | 1.03±0.47 | 1.02±0.21 | 1.07±0.09 | 1.09±0.17 |
| kyotorphin | 467.17 | 23.79 | 73 | 3.39±0.39 | 2.37±0.47 | 1.52±0.61 | 1.36±0.36 | 1.16±0.56 | 1.06±0.6 |
| arbutin | 571.90 | 23.867 | 73 | 15.22±1.44 | 7.46±1.29 | 4.52±0.99 | 2.85±0.87 | 1.84±0.91 | 2.65±0.27 |
| 1-Monopalmitin | 258.08 | 24.136 | 73 | 2.79±1.59 | 2.06±1.08 | 1.52±0.48 | 2.35±1.98 | 1.25±0.43 | 0.86±0.42 |
| sucrose | 797.50 | 24.388 | 73 | 649.35±22.42 | 113.88±11.99 | 68.58±11.56 | 23.95±2.78 | 13.05±3.43 | 6.56±0.94 |
| lactulose | 653.39 | 24.754 | 204 | 1.18±0.59 | 1.06±0.58 | 0.78±0.17 | 0.73±0.26 | 0.77±0.14 | 0.84±0.15 |
| lactose | 644.65 | 24.948 | 73 | 70.01±80.68 | 5.29±2.74 | 6.10±6.2 | 2.50±2.59 | 0.00 | 0.00 |
| cellobiose | 881.18 | 25.091 | 160 | 36.65±26.2 | 16.18±0.98 | 14.14±1.06 | 14.62±1.27 | 13.85±1.28 | 13.93±2.56 |
| maltose | 904.62 | 25.158 | 191 | 241.05±150.76 | 145.28±4.43 | 52.47±3.38 | 14.95±0.63 | 12.04±0.89 | 10.83±2.14 |
| trehalose | 915.86 | 25.200 | 204 | 303.96±132.59 | 275.21±12.51 | 213.61±12.39 | 205.04±11.72 | 187.17±11.17 | 195.76±19.28 |
| Sophorose | 834.83 | 25.640 | 204 | 134.96±3.97 | 8.36±0.40 | 6.1±0.32 | 4.99±0.22 | 7.48±0.42 | 7.49±0.59 |
| Gentiobiose | 891.12 | 25.714 | 204 | 24.57±1.12 | 19.75±0.85 | 16.82±1.14 | 14.94±0.8 | 21.88±1.54 | 23.18±3.32 |
| melibiose | 829.43 | 26.026 | 204 | 5.56±0.51 | 1.78±0.34 | 2.15±0.26 | 3.64±0.21 | 2.07±0.41 | 2.31±0.21 |
| Isomaltose | 922.19 | 26.090 | 281 | 40.53±2.14 | 109.73±4.81 | 91.97±7.81 | 94.67±6.23 | 122.62±12.58 | 176.94±14.08 |
| 4-Androsten-19-ol-3,17-dione | 209.36 | 26.326 | 355 | 1.5±0.21 | 0.07±0.09 | 0.00 | 0.00 | 0.00 | 0.00 |
| Epigallocatechin | 421.70 | 26.393 | 204 | 0.85±0.11 | 0.00 | 0.00 | 0.00 | 0.00 | 0.00 |
| Galactinol | 529.39 | 26.466 | 73 | 2.40±0.19 | 0.39±0.17 | 0.40±0.19 | 0.52±0.27 | 0.55±0.07 | 0.55±0.31 |
| raffinose | 745.67 | 30.186 | 204 | 53.57±5.95 | 23.51±2.45 | 14.27±2.64 | 3.53±0.39 | 9.66±1.12 | 7.27±0.84 |
| maltotriose | 747.88 | 32.190 | 204 | 18.61±2.2 | 10.83±0.71 | 2.66±0.23 | 0.00 | 0.00 | 0.00 |
| prunin degr. Prod. | 539.69 | 34.507 | 204 | 4.83±2.01 | 1.65±0.11 | 1.44±0.66 | 1.03±0.77 | 0.35±0.23 | 0.28±0.35 |

**Supplemental Table 2 The relative concentrations of differential metabolites during fermentation of black waxy rice wine**

| compound | 2d | 4d | 6d | 11d | 17d | 24d | *P* | VIP |
| --- | --- | --- | --- | --- | --- | --- | --- | --- |
| Lactic acid | 3776.64±129.57 | 2703.81±363.69 | 2273.52±190.14 | 2389.72±87.18 | 2412.16±178.16 | 2439.96±118.62 | <0.01 | 6.3461 |
| N-cyclohexylformamide | 105.3±4.98 | 156.61±6.93 | 135.23±18.41 | 155.62±12.09 | 153.61±7.51 | 147.44±8.50 | <0.01 | 1.4565 |
| Succinic acid | 567.72±24.84 | 254.4±11.95 | 225.68±18.15 | 248.11±13.74 | 235.98±14.08 | 252.1±11.69 | <0.01 | 3.6148 |
| Alanine | 3.86±0.40 | 13.46±2.85 | 14.14±0.79 | 17.42±1.47 | 16.64±3.15 | 18.87±1.82 | <0.01 | 1.0775 |
| Itaconic acid | 7.84±0.73 | 13.83±3.20 | 12.3±1.50 | 17.94±4.69 | 19.34±2.19 | 19.92±0.73 | 0.028 | 1.4081 |
| 2,4-Diaminobutyric acid | 39.81±4.18 | 34.06±4.48 | 30.38±0.33 | 35.94±2.40 | 35.6±3.87 | 36.44±3.31 | <0.01 | 1.3909 |
| Conduritol b epoxide | 61.75±10.58 | 32.11±1.27 | 23.16±1.47 | 30.01±4.84 | 34.4±5.47 | 33.77±2.28 | <0.01 | 1.0481 |
| Maltotriose | 18.61±2.20 | 10.83±0.77 | 2.66±0.23 | 0.00 | 0.00 | 0.00 | <0.01 | 1.1028 |
| Pyruvic acid | 2.69±0.93 | 16.37±0.98 | 14.08±1.56 | 1.85±0.17 | 12.92±1.02 | 14.07±0.93 | <0.01 | 2.3453 |
| L-Malic acid | 51.99±4.12 | 26.7±1.84 | 24.09±2.40 | 20.05±1.86 | 25.56±3.70 | 27.06±2.97 | <0.01 | 3.6148 |
| Glucose-1-phosphate | 988.06±237.32 | 351.16±24.83 | 288.58±38.83 | 334.32±56.22 | 463.65±10.17 | 431.51±35.38 | <0.01 | 4.9029 |
| α-D-glucosamine 1-phosphate | 29.52±13.38 | 17.95±0.48 | 16.68±0.98 | 16.87±0.63 | 22.76±1.49 | 24.16±1.33 | <0.01 | 1.5179 |
| Phosphate | 481.37±41.84 | 354.69±9.03 | 345.7±21.23 | 362.25±17.16 | 376.36±21.81 | 419.22±29.74 | <0.01 | 2.2105 |
| Stearic acid | 85.19±5.85 | 119.86±14.68 | 106.59±14.05 | 108.17±11.41 | 107.07±7.99 | 96.37±9.67 | <0.01 | 1.1229 |
| 2-Deoxyerythritol | 50.17±2.62 | 32.21±1.26 | 27.45±2.42 | 22.17±1.2 | 26.12±2.29 | 29.81±1.25 | 0.015 | 1.6284 |
| Sophorose | 134.96±3.97 | 8.36±0.44 | 6.1±0.32 | 4.99±0.22 | 7.48±0.42 | 7.49±0.59 | <0.01 | 2.3046 |
| Fructose | 0.00 | 24.87±1.18 | 23.21±1.85 | 17.32±0.99 | 26.93±2.46 | 26.73±12.11 | <0.01 | 1.0207 |
| Mannose | 16.24±6.99 | 136.58±63.27 | 172.86±11.54 | 184.91±4.16 | 166.05±12.35 | 161.48±13.5 | <0.01 | 2.0479 |
| Glucose | 2.63±0.44 | 2.36±0.26 | 1.47±0.69 | 0.57±0.25 | 8.22±1.49 | 6.11±1.11 | <0.01 | 1.7612 |
| Maltose | 241.05±150.76 | 145.28±4.78 | 52.47±3.38 | 14.95±0.63 | 12.04±0.89 | 10.83±2.14 | <0.01 | 4.5042 |
| Trehalose | 303.96±132.59 | 275.21±13.51 | 213.61±12.39 | 205.04±11.72 | 187.17±11.17 | 195.76±19.28 | <0.01 | 3.5971 |
| Gentiobiose | 24.57±1.12 | 19.75±0.92 | 16.82±1.14 | 14.94±0.80 | 21.88±1.54 | 23.18±3.32 | <0.01 | 1.6802 |
| Glycine | 16.3±2.18 | 24.71±0.79 | 22.8±1.70 | 26.52±1.30 | 25.52±1.22 | 28.11±1.33 | <0.01 | 1.1445 |
| Uracil | 0.00 | 4.84±0.41 | 3.76±1.71 | 3.29±2.33 | 4.63±0.61 | 0.00 | <0.01 | 1.5013 |
| Aminomalonic acid | 14.88±1.10 | 10.03±1.25 | 9.07±0.76 | 7.79±0.82 | 8.52±1.67 | 6.37±0.66 | 0.024 | 1.0104 |
| Proline | 58.09±4.49 | 54.04±4.01 | 54.08±4.46 | 52.75±3.83 | 57.85±3.82 | 61.30±1.67 | 0.036 | 1.2439 |
| Maleamate | 43.68±3.70 | 24.86±1.46 | 23.4±1.94 | 26.17±1.72 | 25.94±2.88 | 31.08±1.11 | <0.01 | 1.6470 |
| Glutamine | 15.5±2.15 | 62.65±11.9 | 55.05±11.34 | 50.77±6.50 | 60.23±2.76 | 42.69±4.04 | <0.01 | 1.3528 |
| 3,6-Anhydro-D-galactose | 7.57±5.84 | 35.43±1.56 | 33.49±1.68 | 29.58±1.9 | 34.07±2.18 | 37.15±1.92 | <0.01 | 1.0646 |
| 2-Deoxy-D-galactose | 96.45±67.34 | 7.95±0.83 | 9.44±3.40 | 14.22±4.27 | 13.63±1.79 | 21.37±4.35 | 0.015 | 1.6284 |
| Adipamide | 31.95±20.72 | 3.52±1.26 | 4.04±1.17 | 5.23±1.88 | 3.96±1.62 | 7.83±2.96 | 0.028 | 1.1131 |
| Citric acid | 2.85±4.05 | 42.39±1.99 | 37.22±3.89 | 19.58±1.21 | 39.28±2.18 | 43.36±2.68 | <0.01 | 1.2837 |
| 1,5-Anhydroglucitol | 43.69±30.13 | 15.8±3.00 | 13.37±1.4 | 13.85±3.11 | 20.58±2.42 | 16.21±1.13 | <0.01 | 1.3564 |
| Sorbitol | 56.09±4.50 | 38.45±1.03 | 35.96±2.46 | 39.89±1.36 | 53.04±2.22 | 59.57±2.59 | 0.011 | 1.1547 |
| Myo-inositol | 10.58±0.30 | 31.37±0.62 | 28.79±2.18 | 31.98±1.41 | 45.17±1.87 | 57.84±2.5 | <0.01 | 2.3334 |
| D-Glucoheptose | 27.62±7.43 | 16.97±1.57 | 14.01±4.04 | 13.35±6.08 | 24.22±5.18 | 7.88±1.34 | 0.012 | 1.8643 |
| Glutathione | 0.25±0.19 | 2.06±0.14 | 2.28±0.33 | 4.79±0.36 | 3.22±0.27 | 1.28±0.25 | <0.01 | 1.0643 |
| Sucrose | 649.35±22.42 | 113.88±12.95 | 68.58±11.56 | 23.95±2.78 | 13.05±3.43 | 6.56±0.94 | <0.01 | 4.7399 |
| Isomaltose | 40.53±2.14 | 109.73±5.2 | 91.97±7.81 | 94.67±6.23 | 122.62±12.58 | 176.94±14.08 | <0.01 | 1.6974 |
| Raffinose | 53.57±5.95 | 23.51±2.64 | 14.27±2.64 | 3.53±0.39 | 9.66±1.12 | 7.27±0.84 | <0.01 | 1.0966 |

**Supplemental Table 3 Metabolic pathways identified from the differential metabolites**

|  | Total | Hits | Expected | *p* | log(p) | Holmp | FDR | Impact |
| --- | --- | --- | --- | --- | --- | --- | --- | --- |
| Glutathione metabolism | 23 | 2 | 0.81 | 0.19 | 1.66 | 1 | 1 | 0.43 |
| Galactose metabolism | 17 | 4 | 0.60 | <0.01 | 6.15 | 0.16 | 0.07 | 0.36 |
| Pyruvate metabolism | 23 | 3 | 0.81 | 0.04 | 3.15 | 1 | 0.39 | 0.30 |
| Starch and sucrose metabolism | 18 | 6 | 0.63 | <0.01 | 11.09 | <0.01 | <0.01 | 0.25 |
| Alanine, aspartate and glutamate metabolism | 20 | 4 | 0.70 | <0.01 | 5.51 | 0.25 | 0.07 | 0.24 |
| Citrate cycle (TCA cycle) | 20 | 4 | 0.70 | <0.01 | 5.51 | 0.25 | 0.07 | 0.23 |
| Glyoxylate and dicarboxylate metabolism | 14 | 3 | 0.49 | 0.01 | 4.52 | 0.67 | 0.14 | 0.22 |
| Glycine, serine and threonine metabolism | 26 | 2 | 0.91 | 0.23 | 1.47 | 1 | 1 | 0.21 |
| Inositol phosphate metabolism | 19 | 1 | 0.67 | 0.50 | 0.70 | 1 | 1 | 0.16 |
| Amino sugar and nucleotide sugar metabolism | 24 | 3 | 0.84 | 0.05 | 3.04 | 1 | 0.37 | 0.14 |
| Glycolysis or Gluconeogenesis | 24 | 2 | 0.84 | 0.20 | 1.59 | 1 | 1 | 0.10 |
| Pyrimidine metabolism | 35 | 2 | 1.23 | 0.35 | 1.05 | 1 | 1 | 0.06 |
| Fructose and mannose metabolism | 17 | 2 | 0.60 | 0.12 | 2.15 | 1 | 0.76 | 0.05 |
| Nitrogen metabolism | 8 | 2 | 0.28 | 0.03 | 3.53 | 1 | 0.32 | 0 |
| Butanoate metabolism | 17 | 2 | 0.60 | 0.12 | 2.15 | 1 | 0.76 | 0 |
| Cyanoamino acid metabolism | 10 | 1 | 0.35 | 0.30 | 1.20 | 1 | 1 | 0 |
| Methane metabolism | 11 | 1 | 0.39 | 0.33 | 1.12 | 1 | 1 | 0 |
| Pentose and glucuronate interconversions | 12 | 1 | 0.42 | 0.35 | 1.05 | 1 | 1 | 0 |
| Propanoate metabolism | 14 | 1 | 0.49 | 0.40 | 0.93 | 1 | 1 | 0 |
| Aminoacyl-tRNA biosynthesis | 67 | 3 | 2.35 | 0.42 | 0.86 | 1 | 1 | 0 |
| Pantothenate and CoA biosynthesis | 16 | 1 | 0.56 | 0.44 | 0.83 | 1 | 1 | 0 |
| Tyrosine metabolism | 19 | 1 | 0.67 | 0.50 | 0.70 | 1 | 1 | 0 |
| Porphyrin and chlorophyll metabolism | 20 | 1 | 0.70 | 0.51 | 0.67 | 1 | 1 | 0 |
| Valine, leucine and isoleucine biosynthesis | 24 | 1 | 0.84 | 0.58 | 0.54 | 1 | 1 | 0 |
| Cysteine and methionine metabolism | 33 | 1 | 1.16 | 0.70 | 0.36 | 1 | 1 | 0 |
| Arginine and proline metabolism | 37 | 1 | 1.30 | 0.74 | 0.30 | 1 | 1 | 0 |
| Biosynthesis of unsaturated fatty acids | 42 | 1 | 1.47 | 0.78 | 0.24 | 1 | 1 | 0 |
| Purine metabolism | 60 | 1 | 2.10 | 0.89 | 0.12 | 1 | 1 | 0 |
